# Diagnostic Differentiation Between Two Rare Entities—Metastatic Peritoneal Mesothelioma With Duodenal Involvement and Duodenal Synovial Sarcoma: A Case Report

**DOI:** 10.7759/cureus.74764

**Published:** 2024-11-29

**Authors:** Bruna Haueisen Figueiredo Zwetkoff, Fauze Maluf-Filho, Filadélfio E Venco, Roberto Blasbalg, Evandro Sobroza de Mello, Mauricio Fernando de Almeida Barros

**Affiliations:** 1 Department of Endoscopy, Hospital Moriah, São Paulo, BRA; 2 Department of Gastroenterology, Cancer Institute, University of São Paulo, São Paulo, BRA; 3 Department of Pathology, Fila Medicina e Patologia, São Paulo, BRA; 4 Department of Image Diagnostic, Hospital Moriah, São Paulo, BRA; 5 Department of Pathology, University of São Paulo, São Paulo, BRA; 6 Department of Pathology, Hospital Alemão Oswaldo Cruz, Laboratório Centro de Imuno-histoquímica, Citopatologia e Anatomia Patológica (CICAP), São Paulo, BRA; 7 Department of Gastroenterology, Barros e Zaidan - Fígado e Gastro Cirurgia e Clínica, São Paulo, BRA

**Keywords:** case report, endoscopic ultrasound-guided biopsy, fish test, peritoneal mesothelioma, synovial sarcoma

## Abstract

This case report highlights the diagnostic challenges in distinguishing between metastatic peritoneal mesothelioma with duodenal involvement and synovial sarcoma of the duodenum, two rare and complex entities. A 59-year-old woman presented with nonspecific abdominal symptoms, and imaging revealed a heterogeneous lesion between the right hepatic lobe and duodenum. Endoscopic ultrasound-guided biopsy and subsequent histopathological analysis initially suggested synovial sarcoma, but further examination, including a FISH assay, confirmed the diagnosis of malignant peritoneal mesothelioma. This case underscores the importance of integrating detailed medical history, imaging, and advanced diagnostic techniques to achieve an accurate diagnosis in rare conditions. Early and precise identification of such diseases is crucial for appropriate therapeutic management and has significant implications for patient prognosis.

## Introduction

This case report highlights the diagnostic challenges encountered in distinguishing between two rare entities: metastatic peritoneal mesothelioma with duodenal involvement and synovial sarcoma of the duodenum [1,2]. We underscore the importance of combining a detailed medical history, imaging findings, and histopathological results to improve diagnostic accuracy, including the role of endoscopic ultrasound (EUS)-guided tissue acquisition in the evaluation of complex and unusual cases.

## Case presentation

**Figure 1:**
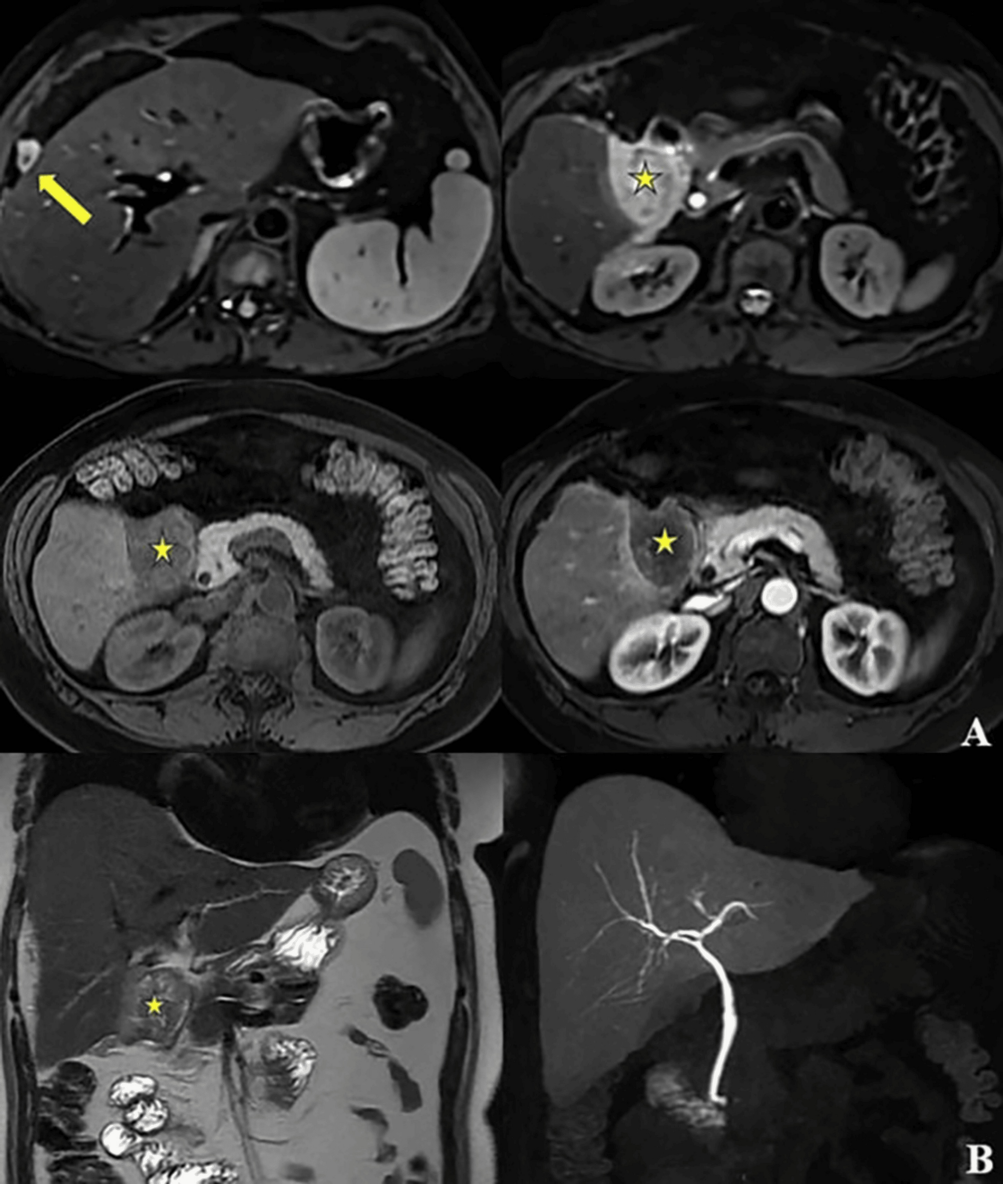
Magnetic resonance imaging revealing a 6.0 x 5.0 cm heterogeneous lesion between the right hepatic lobe and duodenum (star), with unclear margins and hypovascular pattern, featuring small nodules near the diaphragm (arrow). (A) Axial plane. (B) Coronal plane.

A 59-year-old oligosymptomatic woman with no history of asbestos exposure presented with a nonspecific condition of abdominal pain and discomfort, accompanied by bloating and abdominal distension for approximately 60 days. In accordance with the policy of the institutional research ethics committee, case reports are exempt from research ethics committee approval. Written informed permission was obtained from the patient to publish the case report and images for scientific purposes. During routine diagnostics, a magnetic resonance imaging (MRI) scan showed a heterogeneous expansive lesion between the right hepatic lobe and duodenum, with partially defined margins, measuring approximately 6.0 x 5.0 cm. The lesion exhibited a hypovascular pattern and was predominantly peripheral to the contrast medium, with several small nodules interpositioned between the surface of the right hepatic lobe and diaphragm (Figure 1).

**Figure 2:**
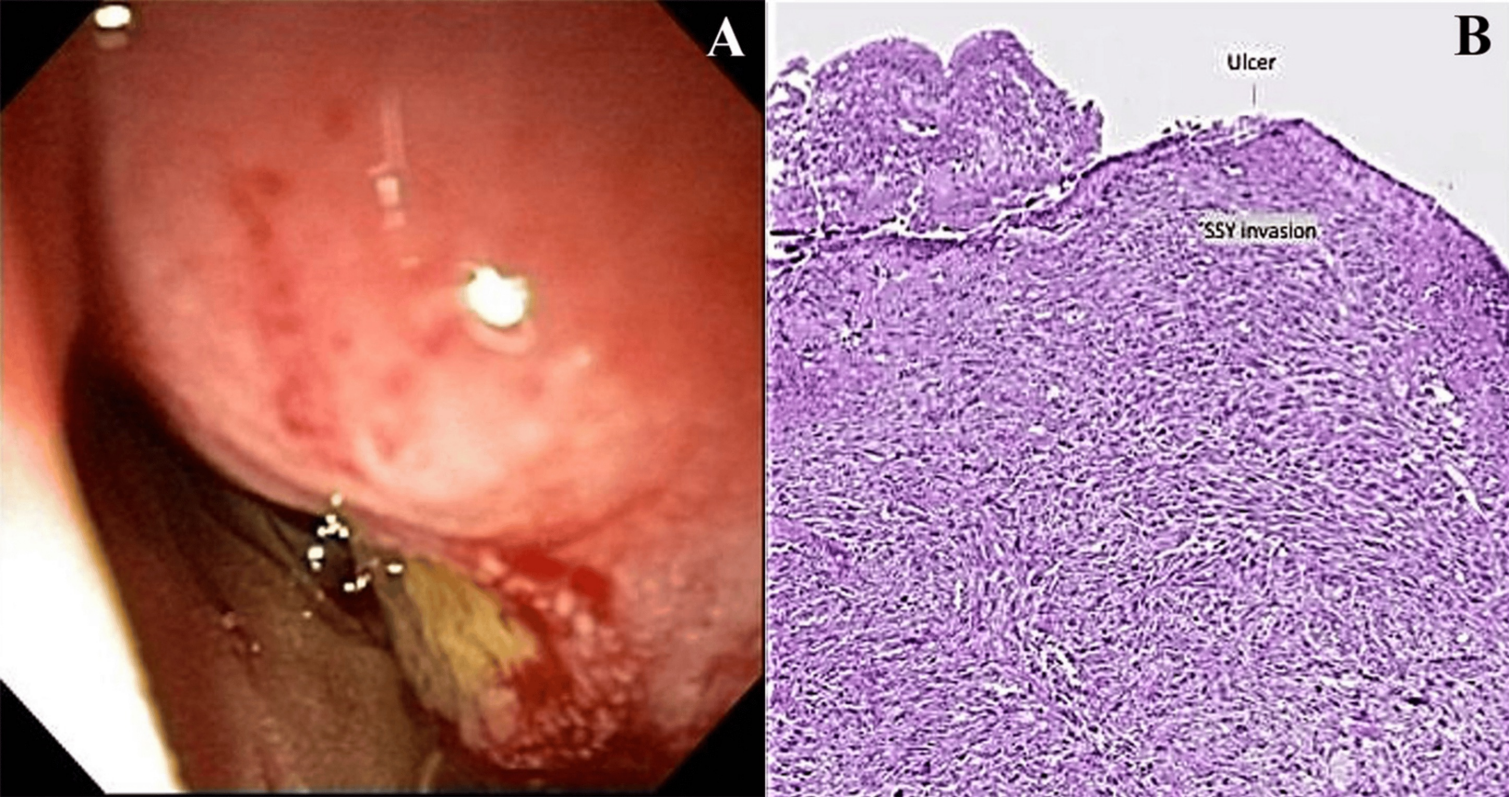
(A) Duodenal ulcer under endoscopic visualization. (B) Histopathology revealing mural invasion.

Further investigation with colonoscopy and upper gastrointestinal endoscopy revealed bulging of the lower wall of the second portion of the duodenum, extending to the descending duodenum, where an extensive ulcerated area with clear and regular margins was observed (Figure 2).

In light of these findings, EUS was performed. EUS images revealed a hypoechoic, hypovascular, heterogeneous lesion with clear and irregular margins, measuring 6.0 x 4.0 cm, infiltrating the duodenal wall and associated with adjacent lymphadenopathy. EUS-guided fine needle aspiration and core biopsy as well as endoscopic biopsies of the ulcer margins were conducted.

**Figure 3:**
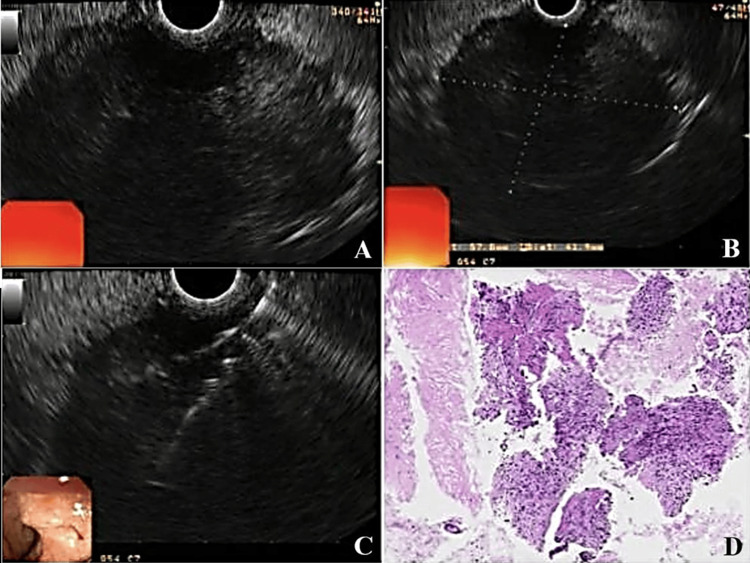
(A and B) Endoscopic ultrasound (EUS) revealing a hypoechoic, hypovascular, heterogeneous lesion with irregular margins infiltrating the duodenal wall and associated with adjacent lymphadenopathy. (C) EUS-guided core biopsy/endoscopic biopsies and (D) histopathology showing scattered anaplastic cells, mucin-containing gland-like structures, and sarcomatoid spindle cells.

**Figure 4:**
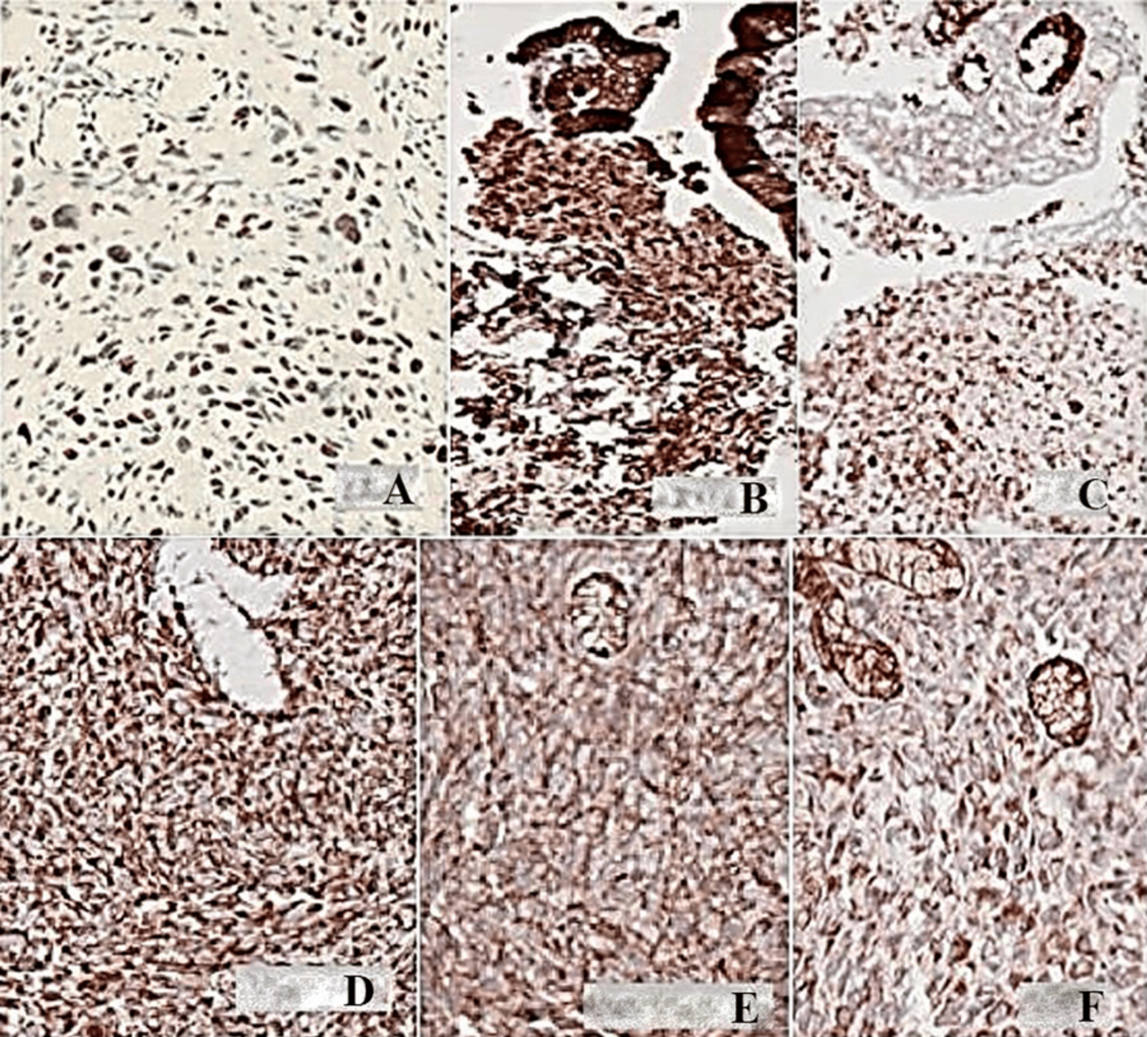
Immunohistochemical panel: (A) Transducin-like enhancer of split 1 (TLE1); (B) AE1/AE3; (C) epithelial membrane antigen (EMA); (D) Vimentin; (E) Beta-catenin; and (F) CD99.

Histopathological analysis revealed epithelium with scattered anaplastic cells and gland-like structures containing mucin, along with sarcomatoid spindle cells (Figure 3). An ancillary immunohistochemical panel was positive for S100, AE1/AE3, epithelial membrane antigen (EMA), vimentin, CD99, beta-catenin, and transducin-like enhancer of split 1 (TLE1). Morphological and immunohistochemical findings were consistent with high-grade biphasic sarcoma, with both spindle cell and epithelioid components, leading to the patient being referred for surgical treatment (Figure 4).

**Figure 5:**
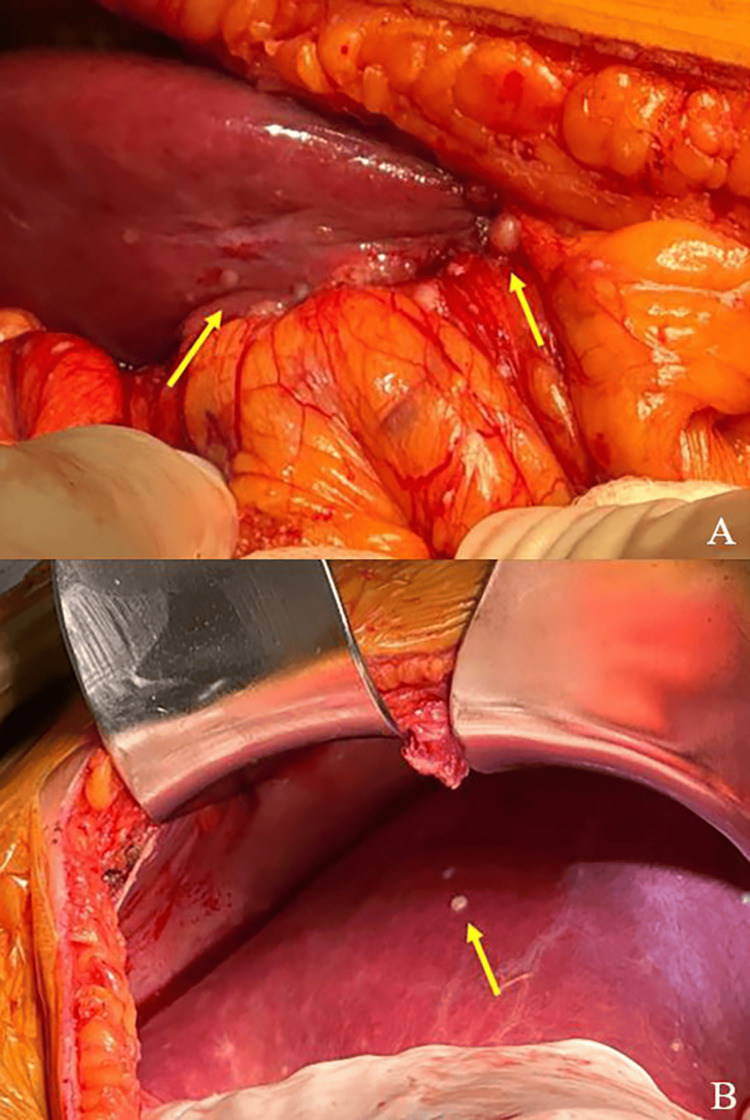
Metastatic implants identified during the surgical procedure (A) in the liver and peritoneum (arrows) and (B) on the liver surface (arrow).

**Figure 6:**
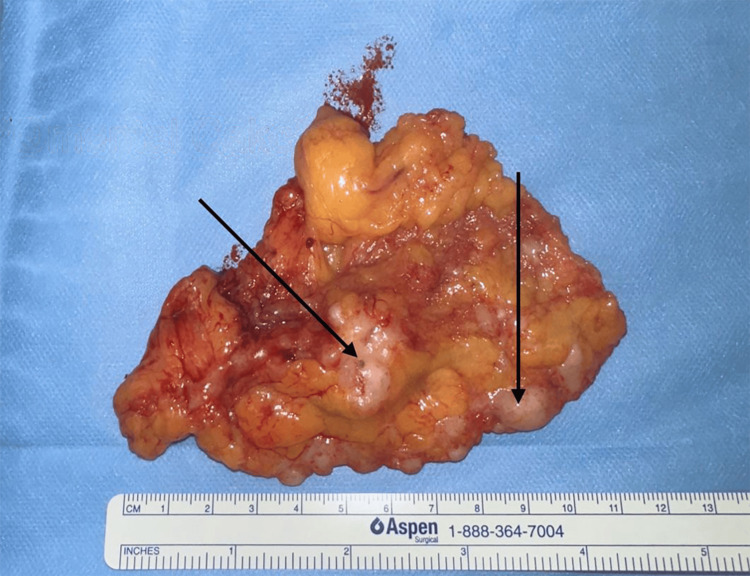
Resected specimen of the omentum revealing the presence of metastatic implants known as ‘omental cake’ (arrows).

A right subcostal laparotomy revealed a deeply entrenched duodenal tumor adherent to the liver, along with several small nodules of peritoneal implants scattered throughout the upper abdominal cavity and in the greater omentum, usually described or characterized as ‘omental cake’ (Figure 5). Therefore, curative surgical resection was no longer possible. A prophylactic gastrojejunostomy was then performed, along with the excision of a few peritoneal and hepatic implants for pathological examination. Segmental omentectomy was performed concomitantly for the same purpose (Figure 6).

**Figure 7:**
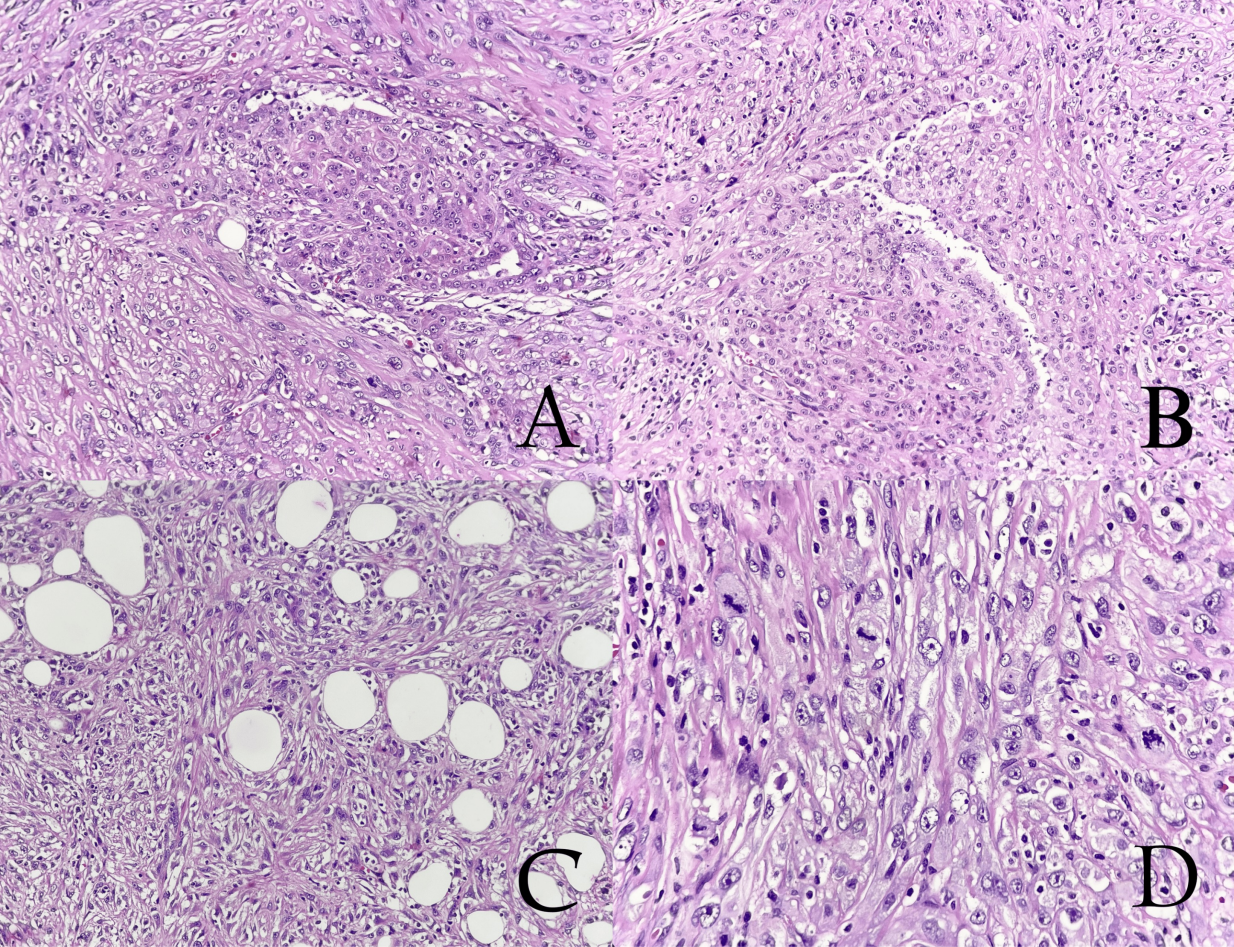
(A and B) A biphasic tumor with epithelial gland-forming component mixed with highly atypical spindle cells. (C) Spindle cell component infiltrating perivisceral fat. (D) Brisk mitotic activity.

**Figure 8:**
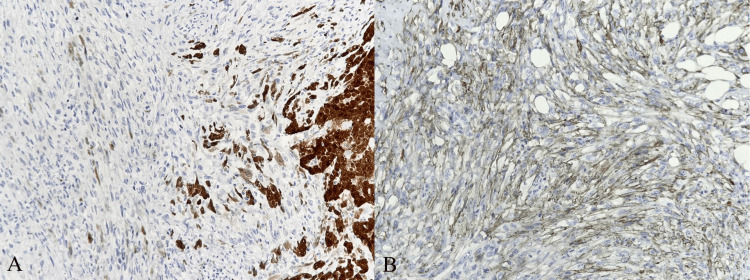
(A) Calretinin strong positivity mostly in epithelioid cells. (B) D2-40 staining in both spindle and epithelioid neoplastic cells.

Histological analysis of the surgical specimens confirmed the presence of a malignant biphasic tumor with a mixed spindle cell and epithelioid pattern infiltrating the peritoneum, with brisk mitotic activity (Figure 7) deviating from previous results due to the recurrent positivity of mesothelial markers, including calretinin, WT-1, and D2-40/podoplanin (Figure 8).

Given the discordant diagnoses, a fluorescence in situ hybridization (FISH) assay was performed to investigate SS18 gene translocation, present in almost all cases of synovial sarcoma. The absence of SS18/SYT gene rearrangement, along with the immunohistochemical panel, confirmed the diagnosis of malignant peritoneal mesothelioma.

The patient was referred for cancer treatment. After treatment failure and unsatisfactory response to three cycles of chemotherapy, checkpoint blockade immunotherapy was initiated and 13 cycles have been performed to date. Surveillance MRI performed three months after diagnosis (June 2024) shows a reduction in the expansive lesion in the right hypochondrium, from 2750cc to 425cc, and the patient remains clinically stable.

## Discussion

Diffuse malignant peritoneal mesothelioma

Mesothelioma is a rare and aggressive neoplasm that arises in areas covered by serous membranes, with the pleura being the most commonly affected anatomical site, followed by the peritoneum, pericardium, and tunica vaginalis. Of the approximately 3300 cases of mesothelioma identified annually in the United States, only 10% to 15% manifest as peritoneal cases [1,3,4]. Generally, the disease is primarily associated with environmental factors, especially prolonged exposure to asbestos, with a latency period of 20 to 40 years [5]. Other risk factors include familial Mediterranean fever, mutations involving BRCA1-associated protein 1 (BAP1), chronic peritonitis, radiation exposure, and simian viruses. However, about 20% to 40% of cases show no predisposing factors, and the disease occurs sporadically [3,5-9].

Peritoneal mesothelioma differs significantly from pleural mesothelioma in presentation, exhibiting an epidemiological profile that is more typical of women (30%-50%) than men (20%-25%) and generally affects patients at a younger age (63 vs 71 years) [6,7]. Symptoms are nonspecific and often related to disease progression, with the development of gastrointestinal tract obstruction and ascites, for example, in advanced cases with disseminated metastatic implants [3,6]. The time interval between symptom onset and disease recognition is estimated to be at least five months [10]. The lack of specific characterization by conventional imaging techniques contributes to the difficulty of diagnosing these lesions. Notably, a more expansive than infiltrative pattern is observed, and the identification of diffuse distributions, ranging from small subcentimeter nodules to larger masses in the abdominal cavity, can be an indirect sign of the disease and may contribute to suspicion. Mesothelioma with diffuse presentation often has a worse prognosis than localized disease [11].

A heterogeneous solid mass of soft tissue with irregular margins is commonly seen on intravenous contrast-enhanced computed tomography (CT) [3,8]. In general, there are no specific imaging or laboratory diagnostic methods that are sufficiently accurate to elucidate the diagnosis, which typically relies on histopathological analysis of surgical specimens, CT-guided biopsy, or laparoscopic biopsy.

Histologically, three variants of the disease can be distinguished. The most common is the epithelioid subtype, which consists mainly of cells resembling normal mesothelial cells with a trabecular pattern and cuboidal or flattened cells having uniform nuclei. The sarcomatoid subtype is less common, but also the most aggressive one, and is typically formed by a compact arrangement of spindle cells [12]. Finally, the mixed subtype accounts for more than 10% of cases and combines components of both epithelioid and sarcomatoid variants [13].

Differentiation between benign and malignant mesotheliomas is often challenging due to the absence of typical features and infrequent mitotic figures. Even with conventional histopathology, the diagnosis of this entity can still be confused with other conditions such as lymphomatosis, sarcoma, adenocarcinoma, peritoneal tuberculosis, and metastatic neoplastic disease [14]. In this context, despite not offering specific antibodies, histology and immunohistochemistry are key tools to determine the disease.

Synovial sarcoma

Synovial sarcoma, formerly known as malignant synovioma, is a rare tumor generally associated with an unfavorable prognosis [2]. Despite the designation ‘synovial sarcoma,’ this tumor is not connected or related to synovium [2]. It accounts for 5% to 10% of all soft tissue sarcomas, with a higher incidence in adolescents and young adults, particularly males [15]. The histogenesis is uncertain, but the main genetic feature is the t(X;18) translocation, present in over 95% of cases, resulting in the fusion of SYT and SSX1 or SSX2 genes [2,16-23]. The specific mechanism by which this gene contributes to the development of synovial sarcoma is not yet understood, but it is believed to be involved in transcriptional activity regulation [23].

This malignant mesenchymal neoplasm occurs primarily in soft tissues, but conceptually it can arise in any anatomical site, with various histological morphologies [18]. Despite well-known histological heterogeneity, there are two main subtypes: monophasic with spindle cells and biphasic with spindle cells and mucin-containing gland-like epithelial structures. In monophasic cases, especially those composed predominantly of spindle cells, the differential diagnosis can be challenging even with immunohistochemistry, and incidence may be underestimated [23]. Approximately 20% of cases may be identified as poorly differentiated and associated with high-grade morphology, often referred to as ‘undifferentiated’ [19]. In 69% of cases, synovial sarcomas are closely associated with joint capsules and tendons in the extremities, affecting intra-abdominal organs in only 1% to 2% of cases [15,18].

The first reliable record of this condition dates back to 1910, when Lejars and Rubens-Duval described a tumor with a biphasic epithelial pattern and spindle cells in the knee joint, which is the most common anatomical site for this lesion [24,25]. Subsequently, with additional studies, the characteristics of the lesions and their ability to develop in anatomical sites other than synovial membranes were accurately defined [2]. These lesions have been reported to occur at multiple sites, such as fallopian tubes, kidneys, prostate, and pharynx [16-18]. Their presence in the gastrointestinal tract is uncommon, and differentiation from other mesenchymal tumors can be challenging, with the esophagus being the site with the highest number of reported cases [26].

Regarding primary synovial sarcoma of the duodenum, there are six published descriptions in the literature and two reports of duodenal metastatic involvement [20-23, 26-29]. In most cases, gastroduodenoscopy reveals an ulcerated lesion extending from the bulb to the third portion of the duodenum, with a case report where no endoscopic abnormalities were detected and the diagnosis was based solely on findings from CT, MRI, and histopathological analysis of the surgical specimen [26].

Diagnosis is challenging and involves differentiation from other gastrointestinal neoplasms, particularly stromal tumors, sarcomas, and lymphomas [20,21]. Imaging in routine diagnostic work-up is often inconclusive and insufficient to reach a conclusive outcome, and it is necessary to rely on histopathological examination of resected specimens for accurate diagnosis.

The importance of differential diagnosis

Despite the availability of advanced diagnostic tools and their reliable performance, such as MRI and EUS-guided tissue sampling for microhistology and immunohistochemistry, obtaining a definitive diagnosis for certain rare conditions remains notably challenging due to their inherently low specificity resulting from infrequent occurrence.

In the case reported here, the biopsy material predominantly showed sarcomatoid features with scattered epithelioid components and positive markers, such as TLE1, initially suggesting a diagnosis of duodenal synovial sarcoma. However, surgical specimen analysis changed the diagnosis because of the heterogeneous nature of the resected specimen, compounded by the FISH assay subsequently performed to investigate the SS18 gene translocation, facilitating a conclusive diagnosis.

Simultaneously, these entities represent two distinct prognostic and treatment paradigms wherein an early and precise diagnosis significantly influences disease progression. Synovial sarcoma has a more favorable prognosis in the pediatric population; in adults, the 5-year survival rate is 50% to 60% [30-32]. Non-metastatic cases are referred for surgical resection with clear margins, and neoadjuvant chemotherapy is associated with improved outcomes particularly in younger patients, but it remains controversial in adults [30]. Radiation therapy may complement surgery or serve as palliative treatment for advanced or metastatic disease. Ongoing clinical trials investigating targeted therapies, such as tyrosine kinase inhibitors, hold promise for future treatment strategies [30].

Conversely, diffuse peritoneal mesothelioma is linked to an even graver prognosis, with a median survival of six to 12 months post-diagnosis [33]. Recent advances have expanded treatment options, encompassing chemotherapy alone and various combinations of cytoreductive surgery, hyperthermic intraperitoneal chemotherapy, immunotherapy, and molecular targeted therapy [33]. Despite encouraging outcomes, the selection of treatment modality remains individualized, underscoring the need for further studies of sufficient robustness to inform broader clinical application.

## Conclusions

Rare conditions such as peritoneal mesothelioma and synovial sarcoma in the gastrointestinal tract pose a major challenge to medical practice. The incorporation of modern and advanced diagnostic methods appears to contribute little to diagnostic elucidation, and even with pathological resources and ancillary methods such as immunohistochemistry, diagnosis can still be challenging. It is important to establish a good correlation between medical history, imaging findings, and histological interpretation, sometimes requiring further in-depth investigation such as the use of FISH testing. An accurate and early diagnosis impacts the institution of appropriate therapy, thereby influencing patients’ prognosis.
